# Mixotrophic culture enhances fucoxanthin production in the haptophyte *Pavlova gyrans*

**DOI:** 10.1007/s00253-024-13199-y

**Published:** 2024-05-31

**Authors:** Erina Yoshida, Yuichi Kato, Akihiko Kanamoto, Akihiko Kondo, Tomohisa Hasunuma

**Affiliations:** 1 Graduate School of Science, Technology, and Innovation, Kobe University, 1-1 Rokkodai, Nada, Kobe, 657-8501 Japan; 2 Engineering Biology Research Center, Kobe University, 1-1 Rokkodai, Nada, Kobe, 657-8501 Japan; 3 OP Bio Factory Co., Ltd, 5-8 Aza-Suzaki, Uruma, 904-2234 Okinawa Japan; 4 RIKEN Center for Sustainable Resource Science, 1-7-22 Suehiro, Tsurumi, Yokohama, 230-0045 Kanagawa Japan

**Keywords:** Fucoxanthin, Marine microalgae, Metabolome analysis, Mixotrophic, *Pavlova gyrans*

## Abstract

**Abstract:**

Fucoxanthin is a versatile substance in the food and pharmaceutical industries owing to its excellent antioxidant and anti-obesity properties. Several microalgae, including the haptophyte *Pavlova* spp., can produce fucoxanthin and are potential industrial fucoxanthin producers, as they lack rigid cell walls, which facilitates fucoxanthin extraction. However, the commercial application of *Pavlova* spp. is limited owing to insufficient biomass production. In this study, we aimed to develop a mixotrophic cultivation method to increase biomass and fucoxanthin production in *Pavlova gyrans* OPMS 30543X. The effects of culturing OPMS 30543X with different organic carbon sources, glycerol concentrations, mixed-nutrient conditions, and light intensities on the consumption of organic carbon sources, biomass production, and fucoxanthin accumulation were analyzed. Several organic carbon sources, such as glycerol, glucose, sucrose, and acetate, were examined, revealing that glycerol was well-consumed by the microalgae. Biomass and fucoxanthin production by OPMS 30543X increased in the presence of 10 mM glycerol compared to that observed without glycerol. Metabolomic analysis revealed higher levels of the metabolites related to the glycolytic, Calvin–Benson–Bassham, and tricarboxylic acid cycles under mixotrophic conditions than under autotrophic conditions. Cultures grown under mixotrophic conditions with a light intensity of 100 µmol photons m^−2^ s^−1^ produced more fucoxanthin than autotrophic cultures. Notably, the amount of fucoxanthin produced (18.9 mg/L) was the highest reported thus far for *Pavlova* species. In conclusion, the use of mixotrophic culture is a promising strategy for increasing fucoxanthin production in *Pavlova* species.

**Key points:**

• *Glycerol enhances biomass and fucoxanthin production in Pavlova gyrans*

• *Metabolite levels increase under mixotrophic conditions*

• *Mixotrophic conditions and medium-light intensity are appropriate for P. gyrans*

## Introduction

Fucoxanthin is an orange-colored carotenoid found in brown macroalgae (*Sargassum*, *Laminaria*, etc.) and several microalgae (*Chrysophyceae* spp. and diatoms) (Sahin et al. 2019). Fucoxanthin plays an important role as a light-harvesting pigment in these algae. Fucoxanthin forms a complex with chlorophyll *a/c* binding protein (Fucoxanthin-chlorophyll *a/c* binding protein complex) that enables harvesting of blue-green light in water and efficiently quenches excess energy. Thus, algae have excellent light-harvesting and photoprotective abilities (Leong et al. 2022).

Fucoxanthin provides several health benefits, such as anti-obesity (Gammone and D’Orazio 2015), anti-cancer (Hosokawa et al. 2004), and anti-inflammatory (Tavares et al. 2020) effects, when ingested by humans. Therefore, fucoxanthin is useful in the food and cosmetics industries. The haptophyte *Pavlova* sp. OPMS 30,543 (OPMS 30,543) produces unsaturated fatty acids, such as eicosapentaenoic acid and docosahexaenoic acid, and is considered an excellent microalga for the biosynthesis of high-value-added bioproducts among fucoxanthin-producing algae (Kanamoto et al. 2021). Furthermore, *Pavlova* species are microalgae lacking cell walls, which facilitates the extraction of fucoxanthin. Culture methods to increase fucoxanthin production from OPMS 30,543 have been developed in previous studies. The highest levels of biomass production and fucoxanthin accumulation in OPMS 30,543 were obtained when the microalgae were cultured using Daigo’s IMK medium (Kanamoto et al. 2021). Fucoxanthin production using *Pavlova gyrans* OPMS 30543X (OPMS 30543X), which is phylogenetically close to OPMS 30,543, was also studied, and it accumulates high levels of fucoxanthin when supplied with NaNO_3_ without any deficiencies (Yoshida et al. 2023).

As described above, the haptophyte *Pavlova* sp. is considered an ideal cell factory for the production of highly functional substances such as fucoxanthin; however, insufficient biomass production limits its application. Insufficient biomass accumulation has also been a bottleneck in diatoms, preventing their commercialization of fucoxanthin production (Marella et al. 2021; Pang et al. 2019). Therefore, to meet the increasing global demand for fucoxanthin, it is essential to establish technologies to improve the production of biomass and metabolites. We hypothesized that mixotrophic growth combining photosynthesis and respiration would enhance biomass production. A previous study reported that mixotrophic and heterotrophic culture conditions can overcome the challenges of growth inhibition in light- and aeration-dependent microalgae (Lee 2001). Patel et al. (2019) found that the advantages of mixotrophic growth include the simultaneous use of the Calvin–Benson–Bassham (CBB) cycle and the pentose phosphate pathway, reduction in biomass loss during the dark phase of light–dark diurnal cycle, and elimination of the oxygen accumulation that leads to oxidative stress. However, few studies have cultured fucoxanthin-producing microalgae in mixotrophic culture, and *Phaeodactylum tricornutum* has been intensively studied as a model species for fucoxanthin-producing microalgae (Marella et al. 2021; Villanova and Spetea 2019). (Marella et al. 2021; Villanova and Spetea 2019). In our previous study, OPMS 30,543 exhibited the highest biomass production (1.79 g dry cell weight [DCW]/L) in a medium containing sodium acetate in a mixed-nutrient culture (Kanamoto et al. 2021). However, the underlying metabolic mechanisms remain unknown.

In this study, we used OPMS 30543X to examine the effects of heterotrophy and mixotrophy on biomass production. In addition, metabolomic analysis was performed to understand the intracellular metabolism in mixotrophs and determine the mixed culture conditions that could efficiently increase biomass production in OPMS 30543X.

## Materials and methods

### Strains and stock cultures

Eukaryotic haptophyte OPMS 30543X (NBRC 102,809, obtained from the National Institute for Biological Resources [NITE] was utilized in this study. The OPMS 30543X medium used in this study was isolated and cultured to be pure and bacteria-free. OPMS 30543X was cultured in double-deck flasks, featuring an upper stage containing the cell culture medium and a lower stage containing KHCO_3_/K_2_CO_3_ solutions to supply 2% CO_2_ gas to the upper stage (Kato et al. 2019). This was performed on a BR-40LF bioshaker (TAITEC, Aichi, Japan) at 30 °C, with cultures under continuous illumination with white, fluorescent light at 50 µmol photons m − ^2^ s − ^1^. The composition of the basic medium was as follows (for 1L): artificial seawater prepared with 19.3 g Marine Art SF-1 (Tomita Seiyaku, Tokushima, Japan) supplemented with 2 × Daigo’s IMK (2IMK; Fujifilm Wako Pure Chemicals, Osaka, Japan), consisting of 400 mg NaNO_3_, 2.8 mg Na_2_HPO_4_, 10 mg K_2_HPO_4_, 5.36 mg NH_4_Cl, 10.4 mg Fe-EDTA, 0.664 mg Mn-EDTA, 74.4 mg Na_2_-EDTA, 0.046 mg ZnSO_4_·7H_2_O, 0.028 mg CoSO_4_·7H_2_O, 0.0146 mg Na_2_MoO_4_·2H_2_O, 0.005 mg CuSO_4_·5H_2_O, 0.0046 mg Na_2_SeO_3_, 0.4 mg Thiamin-HCl, 0.003 mg Biotin, 0.003 mg Vitamin B_12_, and 0.36 mg MnCl_2_·4H_2_O, as previously described (Kanamoto et al. 2021; Yoshida et al. 2023). Seed culture was prepared by subculturing microalgae every 2 weeks at an initial OD_750_ of 0.5. To initiate pre-culture, seed culture was inoculated into double-deck flasks, as previously reported (Yoshida et al. 2023), and cultured for 10 days under continuous white, fluorescent light. For primary or autotrophic culture (control), 70 mL of artificial seawater (19.3 g/L Marine Art SF-1) supplemented with 2IMK and 400 mg/L NaNO_3_, which is equivalent to 2IMK (hereafter referred to as 2 N), was inoculated with microalgae cells from the pre-culture at an initial OD_750_ of 0.5. Cells were cultured in 200 mL baffled flasks at 30 °C, under continuous illumination at 50 µmol photons m^−2^ s^−1^.

### Measurement of biomass

The microalgal culture was harvested in microtubes. Cells were washed with distilled water once and lyophilized in a freeze-dryer FDU-1200 (EYELA, Tokyo, Japan). DCW was calculated by subtracting the weight of the empty microtube from the weight of the microtube with lyophilized cells (Yoshida et al. 2023).

### Measurement of nitrate in the medium

The microalgal culture was harvested in microtubes and centrifugated at 5,000 × *g* for 1 min. The optical density of the supernatant at 220 nm (OD_220_) was measured and the nitrate concentration was calculated using a calibration curve (Collos et al. 1999).

### Analysis of organic carbon concentration in the medium

Residual glycerol, glucose, sucrose, and sodium acetate concentrations in the culture medium were determined using high-performance liquid chromatography (HPLC) (Shimazu, Kyoto, Japan) as previously described (Kumokita et al. 2022). Briefly, the column was maintained at 65 °C, with 5 mM H_2_SO_4_ used as the mobile phase at a flow rate of 0.6 mL/min. Culture supernatants were analyzed using an Aminex HPX- 87 H column (7.8 × 300 mm^2^, 9 μm particle size; Bio-Rad Laboratories, Hercules, CA, USA).

### Pigment analysis

Pigment extraction from lyophilized cells and sample preparation were performed according to previously described methods (Yoshida et al. 2023). Briefly, 700 µL of cell suspension, 300 µL of glass beads, and 500 µL of methanol: acetone :: 1:1 (v/v) solution were added to a microtube, and cells were crushed with an MB1001C (S) multibead shocker (Yasui Kikai, Osaka, Japan). Subsequently, 500 µL of this solution was transferred to a microtube, to which 500 µL of chloroform cooled to 4 °C was added along with 200 µL of ultrapure water for liquid chromatography/mass spectrometry (LC/MS; Fujifilm Wako Pure Chemicals, Osaka, Japan). The sample was subsequently vortexed for 30 s. The supernatant (500 µL) was then transferred to a 3 kDa Amicon Ultra centrifugal filter (Merck Millipore, Burlington, MA, USA) and centrifuged (4 °C, 14,000 *× g*, 90 min) to remove proteins. Carotenoids, such as fucoxanthin, were analyzed and quantified in the samples using an ultra-HPLC system (Waters Corporation, Milford, MA, USA) equipped with a photodiode array (Hasunuma et al. 2019).

### Metabolome analysis

Samples were prepared as previously described (Yoshida et al. 2023). The intracellular metabolite samples were vacuum-dried in a centrifugal evaporator (CEV-3100; EYELA, Tokyo, Japan). Dried samples were redissolved in ultrapure water (20 µL) and analyzed using G7100CE and G6224AA LC/MSD Time-of-Flight systems (Agilent Technologies, Santa Clara, CA, USA), capillary electrophoresis-mass spectrometry (CE-MS). Following previous run conditions (Hsu et al. 2017; Vavricka et al. 2015), metabolites related to glycolysis, the CBB cycle, and the 2-*C*-methylerythritol 4-phosphate (MEP) pathway were analyzed using a 6460 Triple Quad LC/MS (Agilent Technologies) equipped with a Mastro C18 column (2.1 × 150 mm^2^, 3 μm; Shimadzu, Inc.).

### Analysis of photosynthetic activity

Photosynthetic activity was analyzed using the oxygen evolution rate as an indicator following previously described methods (Kim et al. 2020). Briefly, cells were harvested, centrifuged at 8,000 *× g* for 1 min at 4 °C, and resuspended in a solution of the same medium composition as that used for the culture to adjust the cell density to an OD_750_ of 2.0. Cell suspension (1 mL) was added to 10 µL of 0.5 M NaHCO_3_ and then applied to a Clark-type oxygen electrode S1 (Hansatech, King’s Lynn, UK). After acclimation in the dark for 4 min, the oxygen evolution rate was measured after exposing the cells to 200 µmol photons m^−2^ s^−1^ of 660 nm red LED light for 3 min at 30 °C. The oxygen evolution rate was normalized to cell density measured using an automated cell counter TC20™ (Bio-Rad Laboratories).

### Reproducibility and statistical analysis

All data are presented as mean value ± standard deviation (SD) of three replicated experiments. Statistical analysis was performed using Welch’s *t*-test using the R software (version 3.3.3; R Foundation for Statistical Computing, Vienna, Austria).

## Results

### Comparison of organic carbon sources for biomass and fucoxanthin production of OPMS 30543X

**Fig. 1:**
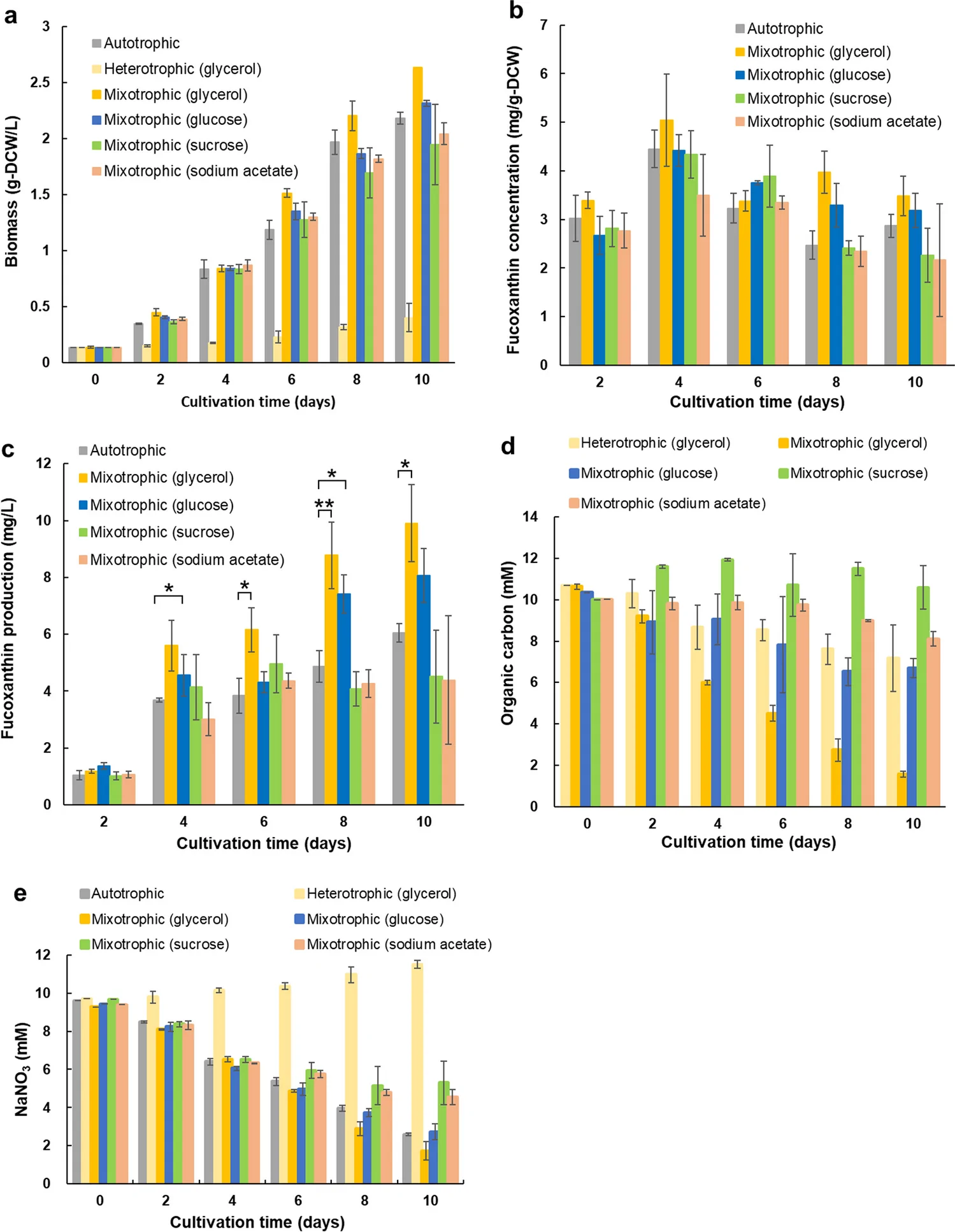
Comparative impact of different organic carbon media used for OPMS 30543X cultivation. **a **Biomass. **b** Fucoxanthin concentration. **c** Fucoxanthin production. **d** Residual organic carbon concentrations in the culture medium. **e** Residual NaNO3 concentration in the culture medium. Tukey’s test determined the statistical significance of results obtained for autotrophic and mixotrophic cultures and a one-way analysis of variance (ANOVA; *p < 0.05, **p < 0.01). Values are presented as mean ± SD from the results of three repeated repetitive experiments

To investigate the effects of different organic carbon sources on biomass and fucoxanthin production, cell growth was analyzed in OPMS 30543X under autotrophic, mixotrophic, and heterotrophic conditions. Autotrophic cells were cultured in 2IMK + 2 N medium, whereas heterotrophic and mixotrophic cells were cultured in 2IMK + 2 N medium with four different organic carbon compounds (glucose, sucrose, glycerol, and sodium acetate) at a concentration of 10 mM. Heterotrophic cultures were grown in the dark, whereas mixotrophic and independent cultures were grown under irradiation at a light intensity of 50 µmol photons m^−2^ s^−1^. All cultures were incubated in triangular flasks maintained at 30 °C and agitated at 100 rpm. The cells were collected every two days, and the DCW was measured at the end of the experiment to determine biomass production. OPMS 30543X cells, grown in autotrophic and various mixotrophic organic carbon media (glycerol, glucose, sucrose, and sodium acetate), continued to increase in biomass for 10 days (Fig. 1a). The biomass of heterotrophic algae was 0.39 g DCW/L on day 10, with an increase of 0.26 g DCW/L in the biomass from the beginning of the culture. Among the various mixotrophic organic carbon media, the highest biomass was obtained in the presence of glycerol (2.63 g DCW/L) after 10 days of cultivation. Fucoxanthin content did not differ significantly between algae incubated in the autotrophic and mixotrophic media (Fig. 1b). In addition, the amount of fucoxanthin produced by OPMS 30543X grown in mixotrophic glycerol (9.9 mg/L; day 10) was higher than that produced by cells grown in autotrophic media (6.0 mg/L; day 10) (Fig. 1c). The concentration of organic carbon source in the glycerol medium decreased to 1.6 mM by day 10 (Fig. 1d). The concentrations of organic carbon sources in the heterotrophic, glucose, sucrose, and sodium acetate media were 7.2, 6.7, 10, and 8.1 mM, respectively. Upon comparing the medium supernatants at days 0 and 10, the NaNO_3_ concentration at day 0 was found to be reduced in glycerol, glucose, sucrose, and sodium acetate media (Fig. 1e). In contrast, upon comparing medium supernatants on days 0 and 10, NaNO_3_ concentrations on day 10 were found to be increased in the heterotrophic medium, probably due to the shedding of dead cells from the medium supernatant. These results suggest that mixotrophic media supplemented with glycerol were the most effective in maximizing biomass and fucoxanthin production in OPMS 30543X.

### Effects of different glycerol concentrations on biomass and fucoxanthin production

**Fig. 2:**
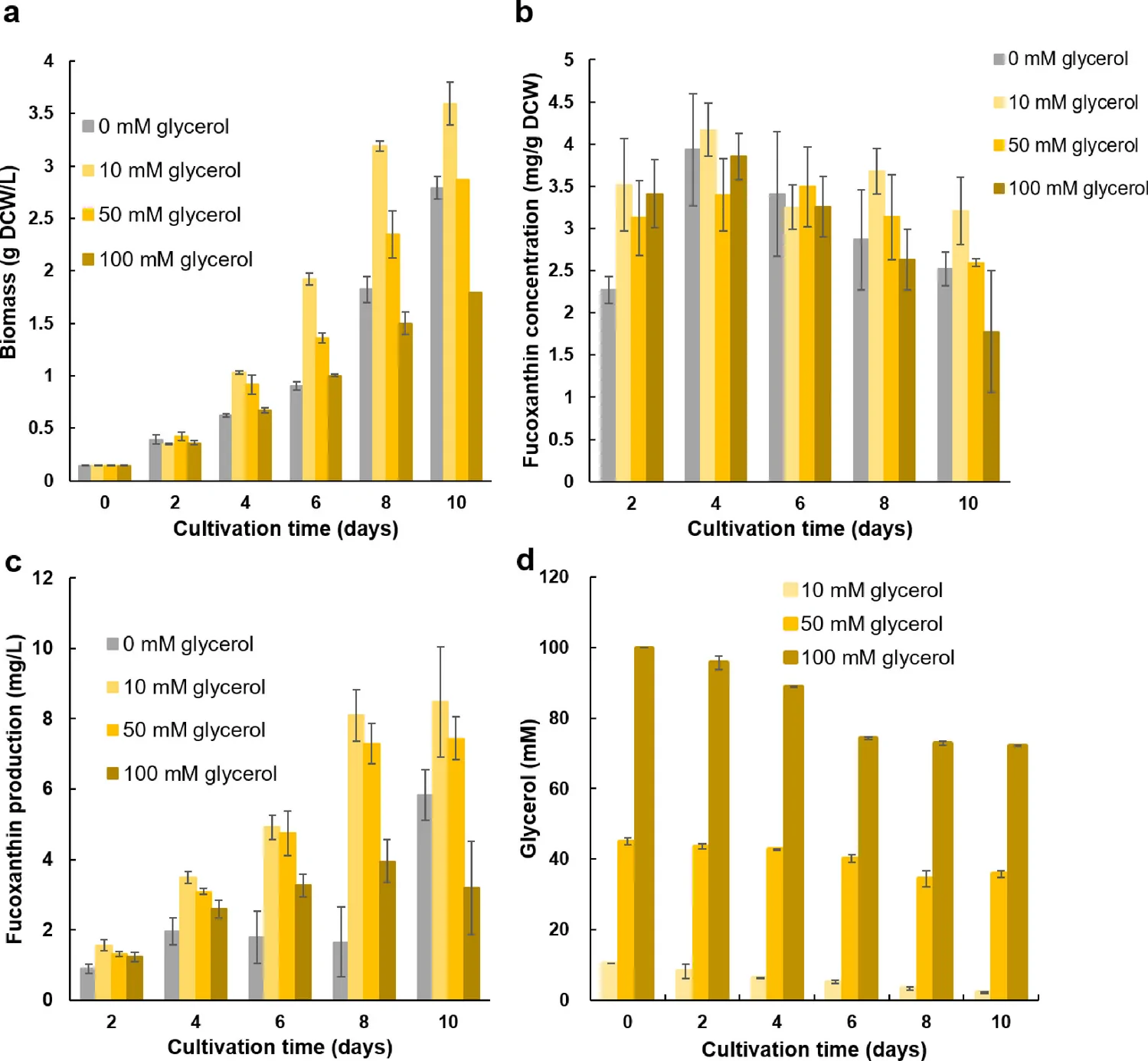
Effects of glycerol concentrations on OPMS 30543X. **a** Biomass. **b** Fucoxanthin concentration. **c** Fucoxanthin production. **d** Residual glycerol concentration in the culture medium. Values are presented as mean ± SD from the results of three repeated repetitive experiments

The results illustrated in Fig. 1 show that the highest amount of fucoxanthin was produced by OPMS 30543X when glycerol was added under mixotrophic conditions. Therefore, the amount of fucoxanthin and biomass produced by OPMS 30543X at different glycerol concentrations (0, 10, 50, and 100 mM) was examined. Cells were collected every two days, and the DCW was measured at the end of the culture period. When cultured with 0, 10, 50, and 100 mM glycerol, the biomass concentrations on day 10 were 2.79, 3.59, 2.86, and 1.79 g DCW/L, respectively (Fig. 2a). Fucoxanthin content was not significantly different among algae cultured at each glycerol concentration (Fig. 2b). The amount of fucoxanthin produced continued to increase in the presence of each concentration of glycerol (0, 10, and 50 mM) over 10 days of culture (Fig. 2c). The maximum amount of fucoxanthin (8.48 mg/L) was produced in the presence of 10 mM glycerol. In the presence of 0, 50, and 100 mM glycerol, different concentrations of fucoxanthin—5.82, 7.44, and 3.18 mg/L, respectively— were produced. This result indicates that biomass concentration and fucoxanthin production decreased when glycerol concentrations in the media increased above 50 mM.

### Metabolite levels in the presence of different types of autotrophic and mixotrophic sources

**Fig. 3:**
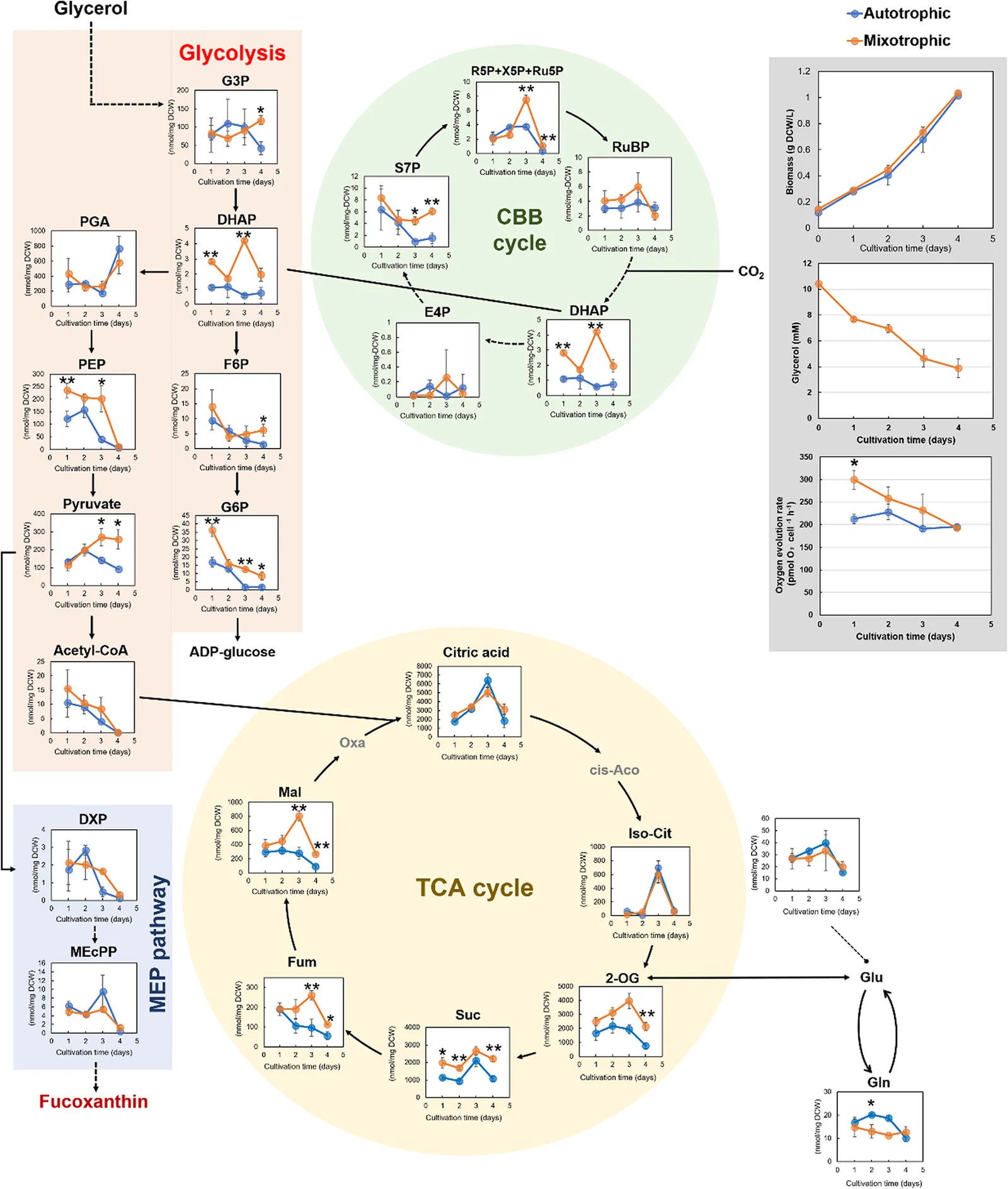
Comparison of the metabolic pool size in OPMS 30543X under autotrophic (blue) and mixotrophic (orange) conditions. Differences in metabolites among the MEP pathway, CBB cycle, glycolysis, TCA cycle, and amino acids. The statistical significance of the results was determined using Welch’s *t*-test (* *p* < 0.05; ** *p* < 0.01). Gray boxes indicate biomass (top), Residual glycerol concentration in the culture medium (middle), and oxygen generation rate (bottom). Values are presented as mean ± SD from the results of three repeated repetitive experiments. S7P, sedoheptulose 7-phosphate; DHAP, dihydroxyacetone phosphate; E4P, erythrose 4-phosphate; RuBP, ribulose-1,5-bisphosphate; R5P + X5P + Ru5P, ribose 5-phosphate + xylulose 5-phosphate + ribulose 5‐phosphate + xylulose 5-phosphate; G3P, glycerol 3‐phosphate; F6P, fructose 6‐phosphate; G6P, glucose 6‐phosphate; PGA, 2-phosphoglycerate + 3-phosphoglycerate; PEP, phosphoenolpyruvate; Oxa, oxaloacetate; Mal, malate; Fum, fumarate; Suc, succinate; 2-OG, 2-ketoglutarate; Iso-Cit, isocitrate; cis-Aco, cis-aconitate; Gln, glutamine; Glu, glutamate; MEcPP, 2-*C*-methyl-d-erythritol-2,4-cyclopyrophosphate; DXP, 1-deoxy-d-xylulose 5-phosphate

When OPMS 30543X cells were cultured under autotrophic (0 mM glycerol) and mixotrophic conditions (10 mM glycerol) and compared, a difference in fucoxanthin production was noted (Fig. 2c). Metabolomic analysis of OPMS 30543X cells cultured on 2IMK + 2 N (autotrophic) and 2IMK + 2 *N* + 10 mM glycerol (mixotrophic) was performed to compare intracellular metabolic pools and identify metabolic mechanisms underlying the association between organic carbon and biomass production. In the CBB cycle, wherein CO_2_-derived carbon is fixed, the levels of sedoheptulose-7-phosphate (S7P), dihydroxyacetone phosphate (DHAP), and ribose 5-phosphate + xylulose 5-phosphate + ribulose 5-phosphate (R5P + X5P + Ru5P) in the mixotrophic group were higher than in the autotrophic group (Fig. 3). In glycolysis, glycerol is synthesized from organic carbon. The levels of glycolysis-related metabolites, such as glycerol 3-phosphate (G3P), DHAP, fructose 6‐phosphate (F6P), glucose 6‐phosphate (G6P), phosphoenolpyruvate (PEP), and pyruvate, in the mixotrophic media, were higher than in the autotrophic media (Fig. 3). The accumulation of metabolites involved in the tricarboxylic acid (TCA) cycle, which include fumarate (Fum), 2-ketoglutarate (2-OG), succinate (Suc), and malate (Mal), was higher under mixotrophic conditions than under autotrophic conditions (Fig. 3). In contrast, glutamine (Gln) was the only metabolite whose levels were higher in autotrophic cultures than in mixotrophic cultures. 1-Deoxy-d-xylulose-5-phosphate (DXP) is located upstream of the MEP pathway (fucoxanthin biosynthesis pathway). No significant differences in DXP levels were found between the autotrophic and mixotrophic conditions.

No significant differences in biomass production were observed between the autotrophic and mixotrophic conditions (p* <0.05). Furthermore, oxygen evolution rates differed between autotrophic and mixotrophic conditions only on day 1 (p* <0.05). After day 2, no differences were observed in oxygen evolution rates between the autotrophic and mixotrophic conditions. This result indicates that the addition of glycerol had a negligible effect on photosynthesis.

### Comparison of the effect of light intensity on biomass and fucoxanthin production

**Fig. 4:**
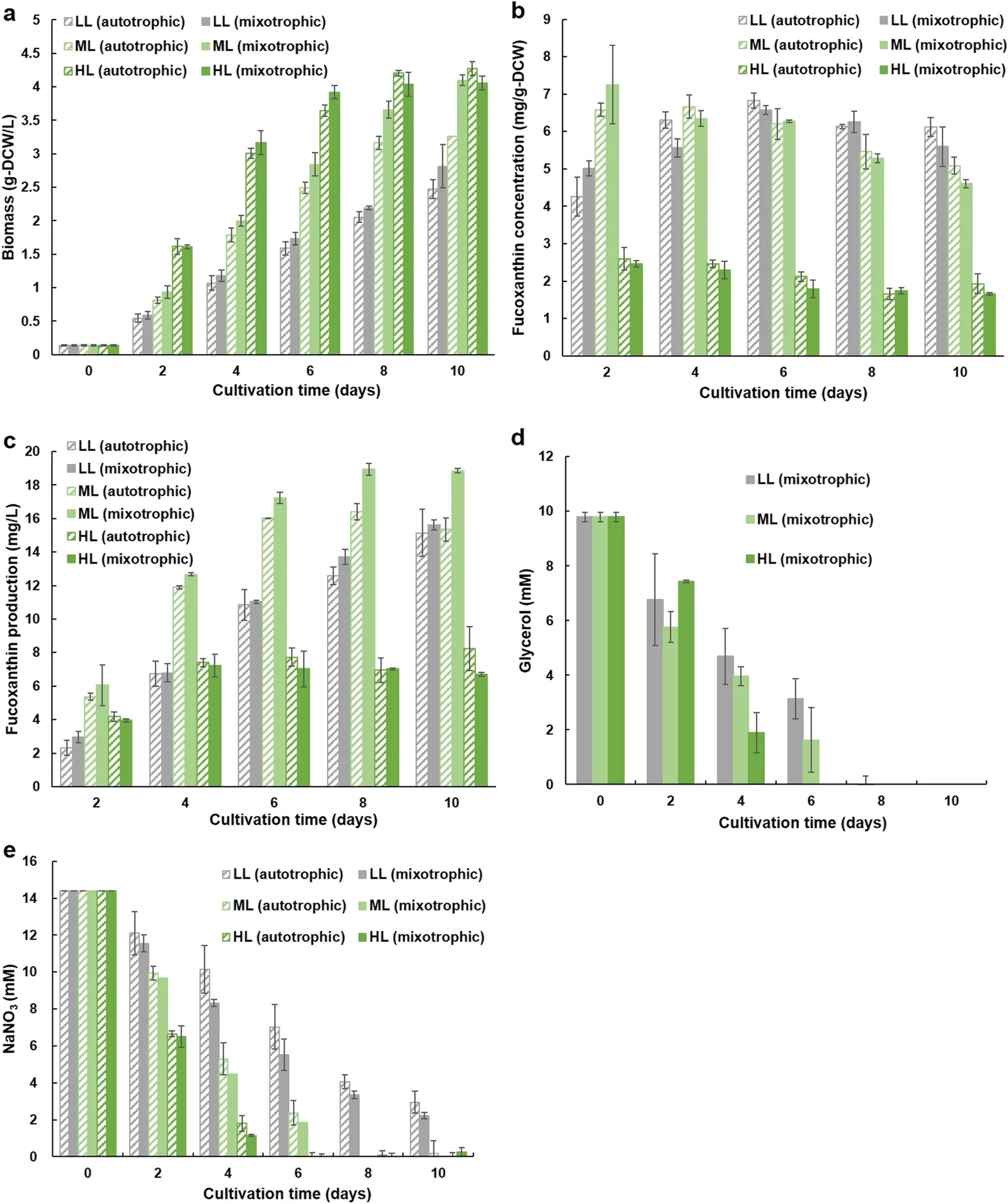
Comparison of light intensities for OPMS 30543X in culture under autotrophic conditions (shaded) and mixotrophic conditions (filled). **a** Biomass. **b** Fucoxanthin concentration (**c**) Fucoxanthin production. (d) Residual glycerol concentration in the culture medium. **e** Residual NaNO_3_ concentration in the culture medium. Low-light (LL), 50 µmol photons m^−2^ s^−1^; medium-light (ML), 100 µmol photons m^−2^ s^−1^; and high-light (HL), 300 µmol photons m^−2^ s^−1^. Values are presented as the average ± SD of results from three replicate experiments

The effects of light intensity on biomass and fucoxanthin production have not been previously studied in OPMS 30543X, and their effects of light intensity on biomass and fucoxanthin production are unknown in the closely related OPMS 30,543. Therefore, the effects of light intensity under mixed-nutrient conditions were investigated. To this end, OPMS 30543X was cultured in 2IMK + 4 N (autotrophic) and 2IMK + 4 *N* + 10 mM glycerol (mixotrophic) media at different light intensities with a 2% CO_2_ supply. Cultures were grown in double-deck flasks, containing 70 mL of the cell culture medium in the upper section and 50 mL of 2 M KHCO_3_/K_2_CO_3_ in the lower section, in the presence of 2% CO_2_ gas under white, fluorescent light adjusted to different light intensities (50, 100, and 300 µmol photons m^−2^ s^−1^). Next, NaNO_3_ (800 mg/L, hereafter referred to as 4 N) equivalent to 4 IMK, was added to the medium to prevent the depletion of nitrogen in the medium, and 1 M HEPES (pH 7.4, adjusted with NaOH) was added to prevent a decrease in the pH (Yoshida et al. 2023). Initially, all culture media were adjusted to pH 7–8. OPMS 30543X was cultured in 2IMK + 4 N (autotrophic) and 2IMK + 4 *N* + 10 mM glycerol (mixotrophic) media. The biomass produced in each flask was calculated by measuring the DCW at the end of the experiment. When cultured under low-light (LL) autotrophic, LL mixotrophic, medium-light (ML) autotrophic, ML mixotrophic, high-light (HL) autotrophic, and HL mixotrophic conditions, the biomass concentrations on day 10 were 2.47, 2.81, 3.26, 4.1, 4.27, and 4.05 g DCW/L, respectively (Fig. 4a). Fucoxanthin concentration varied with light intensity (Fig. 4b). The maximum fucoxanthin concentrations were 6.82 mg/g DCW on day 6 for LL autotrophic, 7.25 mg/g DCW on day 2 for ML mixotrophic, and 3.08 mg/g DCW on day 0 for both HL autotrophic and mixotrophic conditions (3.08 mg/g DCW) (Fig. 4b). No significant differences in fucoxanthin concentrations were observed between autotrophic and mixotrophic algae at the same light intensity. The maximum amounts of fucoxanthin produced under LL autotrophic, LL mixotrophic, ML autotrophic, ML mixotrophic, HL autotrophic, and HL mixotrophic conditions were 15.1 mg/L (day 10), 15.6 mg/L (day 10), 16.3 mg/L (day 8), 18.9 mg/L (day 8), 8.24 mg/L (day 10), and 7.24 mg/g DCW (day 4), respectively (Fig. 4c). Hence, fucoxanthin production was higher in mixotrophic than in autotrophic cultures, even when CO_2_ was supplied. Fucoxanthin production was the highest when OPMS 30543X was cultured under ML mixotrophic conditions. Glycerol depletion occurred with an increase in light intensity, and the glycerol in HL mixotrophic, ML mixotrophic, and LL mixotrophic cultures was completely consumed after 6, 8, and 8 days, respectively (Fig. 2d). NaNO_3_ was completely consumed by day 6 under HL conditions and by day 8 under ML conditions for both autotrophic and mixotrophic conditions (Fig. 4e). In contrast, under LL conditions, NaNO_3_ was not consumed even after 10 days of incubation (Fig. 4e).

## Discussion

The addition of organic carbon sources to the culture medium can stimulate the cell growth of microalgae. The ability of such microalgae to proliferate depends mainly on the species of microalgae, type of organic carbon source, and environmental factors (Chen and Chen 2006; Garcia et al. 2005). Lewin and Lewin (1960) established the growth of diatoms in organic carbon under dark conditions. They reported that the addition of carbon sources, such as glucose, fructose, glycerol, and acetic acid, effectively supported cell proliferation under heterotrophic conditions. Mixotrophic cultures of microalgae have been reported to induce higher growth rates than those induced by autotrophic cultures (Lewin and Hellebust 1970). Initially, *Cyclotella cryptica* was grown in the presence of glucose, and subsequently, the culture of *Navicula saprophila* in the presence of acetic acid yielded promising results (Kitano et al. 1997). Biomass production in the haptophyte *Pavlova lutheri* was enhanced in a mixotrophic modified artificial seawater medium supplemented with 10 mmol sucrose (Bashir et al. 2019). In the presence of sodium acetate, the highest biomass production in OPMS 30,543 cells was observed under mixotrophic conditions (Kanamoto et al. 2021).

The present study provides insight into the effect of mixotrophic culture on increasing biomass and fucoxanthin production in the genus *Pavlova*. Previous studies have explored culture conditions for *Pavlova* sp. strain OPMS 30,543, which is comparable to OPMS 30543X, to maximize biomass production (Kanamoto et al. 2021). In the present study, the investigation of organic carbon source conditions was limited to the examination of carbon types. Carbon concentrations or the fundamental metabolic mechanisms for increasing biomass production have not yet been reported. In the present study, OPMS 30543X cells were cultured under different conditions (type and concentration of organic carbon source), and the effects of carbon source on biomass and fucoxanthin production were compared and investigated. OPMS 30543X showed the highest biomass and fucoxanthin production when glycerol was used as the organic carbon source (Fig. 1a and c). These findings were consistent with the results of a previous study on *P. gyrans* (Ukeles and Rose 1976). In contrast, OPMS 30,543 exhibited the highest biomass production in a medium supplemented with sodium acetate (Kanamoto et al. 2021), whereas *P. lutheri* showed the highest biomass production in a medium supplemented with sucrose (Bashir et al. 2019). These results indicate that different organic carbon sources may enhance biomass production in different haptophyte *Pavlova* species. When using different concentrations of organic carbon sources, biomass production in OPMS 30543X decreased with the addition of > 50 mM glycerol (Fig. 2a). This suggests that high concentrations of glycerol inhibit growth. The decrease in biomass production at high concentrations of organic carbon sources was similar to that observed in *P. lutheri* (Bashir et al. 2019). In OPMS 30543X, sufficient cell proliferation was observed with the addition of very low glycerol concentrations. Therefore, the optimal glycerol concentration for culturing OPMS 30543X cells was determined to be 10 mM. From previous reports, we know that *P. lutheri*, similar to OPMS 30543X, experienced growth inhibition when glycerol concentration was increased. However, *Pavlova* spp. lack a cell wall, rendering them more fragile and osmotic, which may have inhibited their growth. Given that the underlying reason for the observed inhibition cannot be reliably inferred, this phenomenon should be investigated in the future.

In previous studies on biomass production in *Pavlova* species, organic carbon was mostly used under mixotrophic conditions, but these studies could not explain why organic carbon led to an increase in biomass production (Bashir et al. 2019; Kanamoto et al. 2021; Ukeles and Rose 1976). By comparing the intracellular metabolites produced in autotrophic (0 mM glycerol) and mixotrophic (10 mM glycerol) cultures, which showed differences in the extent of biomass production (Figs. 1a and 2a), we determined the metabolites served as bottlenecks in biomass production. This is the first study to investigate metabolic trends regarding the effects of glycerol on biomass accumulation in haptophytes. In microalgae, incorporated glycerol is assimilated into G3P and converted into various metabolites through glycolysis. In mixotrophic OPMS 30543X culture, higher metabolite levels were obtained during glycolysis, in which glycerol uptake occurs, than those observed in cells grown under autotrophic conditions (Fig. 3). In addition, higher metabolite levels were observed in the mixotrophic OPMS 30543X group CBB cycle than in the autotrophic group (Fig. 3). Higher levels of four TCA cycle-related metabolites were also observed under mixotrophic conditions than under autotrophic conditions (Fig. 3). These findings indicate that the addition of glycerol altered metabolism in terms of the accumulation of glycolytic, CBB cycle, and TCA cycle compounds. To demonstrate that glycerol contributes to increased biomass production, verification of the hypothesis that photosynthetic performance is higher under mixotrophic conditions than under autotrophic conditions is necessary. Therefore, we measured the rate of oxygen evolution to evaluate photosynthetic performance and found that the rate of oxygen evolution under mixotrophic conditions was higher than t under autotrophic conditions on day 1, whereas no difference was observed after day 2 (Fig. 3, gray box). Thus, the addition of glycerol had a marginal effect on autotrophic and mixotrophic photosynthesis. The result indicating that mixotrophic media had only a slight effect on photosynthesis is consistent with previous results obtained for the diatoms *Phaeodactylum tricornutum* and *Cylindrotheca* sp. (Villanova et al. 2017; Wang et al. 2023). Thus, the metabolite levels observed in the CBB cycle of the mixotrophic OPMS 30543X group may have been higher than those in the autotrophic group because of the added glycerol effect. These findings suggest that in OPMS 30543X, the accumulation of glycolytic, CBB cycle, and TCA cycle metabolites in mixotrophic cultures is higher than in autotrophic cultures. In other words, mixotrophs may experience bottlenecks in glycolysis, the CBB cycle, and the TCA cycle. HL intensity may increase biomass production because of the flow of accumulated metabolites (especially in the CBB cycle). However, further experiments are required to confirm the reason underlying this phenomenon.

Previous studies on *P. lutheri* have shown that more biomass is produced by mixotrophs than by autotrophs when cultured at appropriate light intensities (Bashir et al. 2019). Moreover, in fucoxanthin-producing microalgae, HL intensity increases biomass production under autotrophic conditions while reducing fucoxanthin content (Wang et al. 2023). To achieve maximum biomass and fucoxanthin production under mixed nutrition conditions, we determined the appropriate light intensity for OPMS 30543X. The highest level of biomass production was obtained under HL intensities, whereas fucoxanthin production was the highest under ML mixotrophic conditions (Fig. 4a and b). The results regarding biomass production in this study differ from the findings of Sloth et al. (2006) and Bashir et al. (2019). Under mixotrophic conditions, they reported that the highest levels of growth were recorded at 100 µmol photons m^−2^ s^−1^ in *Galdieria sulphuraria* and 125 µmol photons m^−2^ s^−1^ in *P. lutheri* (Bashir et al. 2019; Sloth et al. 2006). Furthermore, previous studies have shown that biomass production does not increase at light intensities of 150–210 µmol photons m^−2^ s^−1^, even under autotrophic conditions (McClure et al. 2018). However, the present study showed that OPMS 30543X grew at HL intensities under both autotrophic and mixotrophic conditions. Fucoxanthin production decreased at HL intensities, which is consistent with the results of McClure et al. (2018). This phenomenon has also been observed in *Odontella aurita* (Xia et al. 2013), *Phaeodactylum tricornutum* (Gómez-Loredo et al. 2016), and *Cyclotella cryptica* (Guo et al. 2016). When exposed to strong light, fucoxanthin is converted to diadinoxanthin, resulting in a decrease in fucoxanthin concentration (Bai et al. 2022; Leong et al. 2022). In a previous study on *Pavlova* species, the highest amounts of biomass and fucoxanthin produced were 1.79 g/L and 7.36 mg/L, respectively, under mixotrophic conditions (Kanamoto et al. 2021). Further studies may be needed to elucidate the detailed physiological and metabolic mechanisms of OPMS 30543X under appropriate light intensities and mixotrophic conditions by examining the intracellular pool size of cells incubated under these conditions.

In conclusion, to the best of our knowledge, we achieved the highest amount of fucoxanthin production reported for *Pavlova* species studied to date. The highest amount of fucoxanthin produced in this study was approximately 2.6 times (18.9 mg/L) higher under ML mixotrophic conditions than that reported by Kanamoto et al. (2021); 4.09 g DCW/L of biomass was produced under these conditions. Our findings on the growth of cells under mixed-nutrient conditions and at light intensities that do not inhibit fucoxanthin production may facilitate effective commercial production of fucoxanthin using *P. gyrans* OPMS 30543X.

## Data Availability

The data obtained and/or analyzed in this study are available from the corresponding author upon reasonable request.
